# Effect of drinking water temperature on physiological variables of crossbred dairy cattle at high altitude temperate region of Himalayas

**DOI:** 10.14202/vetworld.2015.1210-1214

**Published:** 2015-10-17

**Authors:** D. M. Golher, P. Thirumurugan, B. H. M. Patel, V. K. Upadhyay, S. Sahu, G. K. Gaur, S. H. Bhoite

**Affiliations:** 1 Division of Temperate Animal Husbandry, Indian Veterinary Research Institute, Mukteshwar, Uttarakhand, India; 2 Livestock Production and Management Section, Indian Veterinary Research Institute, Izatnagar, Bareilly, Uttar Pradesh, India; 3 Department of Animal Husbandry, Livestock Supervisor Shivankhed, Zilla Parishad Latur, Maharashtra, India

**Keywords:** ambient, lactating, relative humidity, ruminal motility, temperate region

## Abstract

**Aim:**

The objective of study was to investigate the effects of drinking water on certain physiological parameters such as heart rate (HR), respiration rate (RR), rectal temperature (RT) and, ruminal motility (RM).

**Materials and Methods:**

The experiment was carried out on 18 farm bred lactating crossbred cows. The animals selected for the study were divided into three groups of six animals each on the basis of milk yield and parity and were allotted to three treatment group of six each such as ambient drinking water temperature at 10.25±0.28°C (ambient water, T_1_), drinking water temperature at 15-20°C (T_2_) and drinking water temperature at 35-40°C (T_3_). All the managemental practices were kept similar during experiment except drinking water temperatures physiological variables such as HR, RR, RT, and RM of the individual cow was measured and recorded twice in a day at 800 h and again at 1400 h two consecutive days in a week 15 min after providing drinking water.

**Result:**

HR and RR at morning and at evening recorded were within the normal physiological level for all the treatment groups. However, RT at morning was comparable in all the treatments whereas at evening it was significantly (p<0.01) higher for cows consuming in T_2_ and in T_3_ than cows consumed (T_1_). The RM during morning among the treatments were non-significant as compared to the rumen motility at evening was significantly higher for (T_1_) and (T_2_) than for cows in (T_3_).

**Conclusion:**

It can be concluded that offering warm drinking water at 35-40°C to crossbred lactating dairy cow is beneficial during winter at high altitude temperate region.

## Introduction

Climate is one of the limiting factors in dairy production as the dairy cows are homeotherms and the maintenance of nearly constant body temperature, when subjected to a wide range of environmental conditions, depends on balancing heat production and heat loss. The geographical and climatological conditions at high mountains are unique. The temperature drops about 0.6°C for every 100 m increase at altitude. Thus, at 4500 m, it should theoretically be 27°C cooler than at sea level [1]. The temperature not only influences thermal comfort, but humidity also plays significant role therefore usually comfort zone is based on temperature and humidity index (THI) or its variation [2]. The limiting values for THI cause heat stress from occurring 70-72. However, in the temperate regions of these values may be lower (68) and higher subtropical and tropical areas (72) [3].

Dairy cows are in the comfort zone when environmental temperatures between 5°C and 25°C [4]. The zone of comfort for Indian cows is 10-26.7°C [5]. Crossbred cattle to the cold environment have not performed same as exotic animals in temperate region because crossbred having about 50% tropical cattle blood which are more adapted to warm climate rather than cold climate [6]. During winter it creates more impact on performance of dairy animals as ambient temperature falls below the lower critical temperature and hence temperature of drinking water also lowers down significantly [7]. At high altitude of the western Himalayas, environmental temperature is very low especially during winter months. The drinking water available to animals of dairy farm through open water troughs is often very cold during these months. Temperature affects the quality directly by changing palatability and acceptance by animal or by disturbing the microflora of digestive tract [8].

Many workers observed that *Bos indicus* cattle differently performed from *Bos taurus* and their crossbred to environmental temperature [6,9]. Therefore, the present study was conducted to explore the utility of different drinking water temperature as an important winter management practice in crossbred cows at high altitude temperate Himalayan region.

## Materials and Methods

### Ethical approval

The research program was conducted after the approval of the Institutional Animal Ethics Committee Indian Veterinary Research Institute (IVRI), Izatnagar, Bareilly, Uttar Pradesh.

### Location and climatic condition

The Experimental Cattle Herd, IVRI, Mukteshwar campus, was the location of the present experiment. Mukteshwar is geographically situated at 79°38’ 52” E longitude, 29°28’ 20” N latitude and 2286 meters altitude above mean sea level on the peak at junction of Gagar and Lohaghat ranges of the Kumaon hill, district Nainital, Uttarakhand state. The annual average temperature during 2006-2009 was 13.89°C with mean monthly temperature of 6.6°C during February and 17.13 59°C during June. Annual average relative humidity (RH) was 61.32% and average annual rainfall was 1509.50 mm [10].

### Experimental animals

The experiment was carried out on eighteen farm bred early to mid-lactating crossbred cows (Holstein Friesian × Haryana) belonging to Experimental Cattle Herd, Division of Temperate Animal Husbandry, IVRI, Mukteshwar campus. The animals selected for the study were divided into three groups of six animals each on the basis of milk yield, parity and body weight. The animals were allotted to three treatment groups (T_1_, T_2_ and T_3_) of six each.

All the experimental animals were housed in tie-barn double row tail-to-tail shed for the entire study period where individual feeding, watering and care were taken. About 1 week adjustment period was provided for all the cows to habituate drinking water in bucket and to standardize watering practices. The observation period was of 8 weeks for each cow in continuation to the adjustment period. All the animals were maintained under uniform feeding and management practices except the differences in temperature of drinking water.

### Treatment-1 (T_1_)

The cows in T_1_ were provided drinking water of ambient temperature 10.25±0.28°C. This is the mean temperature of ambient water recorded at morning and evening watering time during the experiment period.

### Treatment-2 (T_2_)

The T_2_ was offered drinking water at 15-20°C. This is the mean ambient temperature recorded during the summer months from 2006 to 2009 [10]. The 15-20°C water temperature was maintained by adding warm water obtained from hot water supply tank available at dairy farm, and minimum 15°C water temperature was maintained till withdrawal of water.

### Treatment-3 (T_3_)

The group 3 was provided drinking water at 35-40°C. In many experiments, it was reported that during the summer season of tropic cows consumed more warm water (35-40°C) than artificially chilled/cooled water. It was expected that the 35-40°C drinking water temperature provided maximum thermal benefit and palatability to cows during winter months of Mukteshwar climate. The 35-40°C water temperature was maintained by adding ambient water into warm water obtained from hot water supply tank which was available at dairy farm and minimum 35°C water temperature was maintained till withdrawal of water.

### Watering schedule

Drinking water was provided to all the cows in each groups two times a day with scheduled of 800 h (after morning milking) and 1600 h (before evening milking). A measured quantity of *ad* *libitum* water was provided to all animals and adjusting the left over exact consumption of water was recorded for the individual animal. The logic behind such water schedule was to meet the physiological requirements of water during the coldest period of the year.

### Housing and feeding

All the experimental animals were housed in tie-barn double row tail-to-tail shed during the study period. The shed was daily scrubbed and washed with water and disinfectant. The cows under all the treatments were provided with weighed quantity of concentrate feed individually twice daily as per standards. The cows were given 2.0 kg of concentrate daily to meet maintenance requirements. In addition, for every 2.5 kg milk produced, 1.0 kg of concentrate was given. Daily concentrate requirement was worked out every seven days based on average milk production during that period. Roughage obtained from the available resources was fed *ad libitum* to all the cows thrice daily.

### Recording climatic data

Macroclimatic data such as maximum temperature, minimum temperature and RH were collected from weather station of Mukteshwar. Microclimatic data were recorded daily, throughout the experimental periods once in the morning at 800 h and again in the afternoon at 1500 h. This recording was done inside the shed at animal level to quantify the microenvironment prevalent around the animals in all the treatment groups. The climatic variables viz. maximum and minimum temperatures, and dry bulb and wet bulb temperatures were recorded. The psychometric table was used to derive the RH in percentage using the wet and dry bulb readings. The temperature humidity index (THI) was calculated by using equation, THI=0.72 (Tdb+Twb)+40.6 of McDowell [11], where Tdb and Twb are dry bulb and wet bulb temperature, respectively, in °C.

### Physiological variables

Physiological variables like heart rate (HR), respiration rate (RR), rectal temperature (RT) and ruminal motility (RM) of the individual cow was measured and recorded twice in a day at 800 h and again at 1400 h two consecutive days in a week 15 min after providing drinking water. The HR was directly measured by counting for a minute, using a stethoscope and stopwatch. RR was measured by counting the flank movements for two consecutive minutes and then average was taken (breaths/min). Recordings were taken for individual cows with minimum of disturbance. The RT was measured with the help of a clinical thermometer inserted to standard depth and recorded in degree Fahrenheit for each of the cow. The RM was taken in the left paralumbar fossa of the animal by pressing fist up to 3 min for taking the RM reading.

All the above four physiological parameters were recorded in the above sequence on each animal in the same order in which water was provided to the cows. For all the physiological parameters 2 days average was taken to get observations/week for 0800 h and 1400 h onward separately. Full hand dry milking was done in the shed itself in individual tied up system during 530 h and 1530 h. Milkers were kept same up to maximum possible extent and sequence of milking was followed daily to avoid the variation in milking interval of each cow.

### Statistical analysis

The statistical analysis of the experimental data was done as completely randomized design with one-way ANOVA as per Snedecor and Cochran [12]. The means of variables were compared by Duncan’s Multiple Range Test.

## Results and Discussion

### Macroclimatic and microclimatic variables

The range of maximum temperature, minimum temperature, morning RH, and evening RH recorded was 3.0-16.9°C, −2.60-6.80°C, 10-100%, and 26-100%, respectively, for the macro climate. THI value in the morning and evening ranged from 40.60 to 50.39 and 41.46 to 56.30. Similarly, inside the animal shed the range of maximum temperature, minimum temperature, RH and evening RH recorded was 9.0-1.0°C, 0-14°C, 73-89% and 54-91%, respectively. THI in the morning and evening varied from 52.12 to 62.92 and 55.72 to 70.12, respectively. For all these variables, microclimatic changes inside the shed was higher than the outside environment and indicated that tail-to-tail tie-barn house at high altitude temperate region conserved the heat and humidity. This was due to expired air of animals and evaporation of moisture inside the shed. However, optimum productivity of cattle occur at an air temperature of 13-18°C and RH of 60-70%, and lower critical value of THI was 64 [13] (Table-1).

**Table-1:** Macroclimatic and microclimatic variables.

Attributes	Macroclimate	Microclimate
Maximum temperature** (°C)	11.81±0.45	16.61±0.36
Minimum temperature** (°C)	1.52±0.27	6.68±0.61
Mean temperature**(°C)	6.66±0.31	11.64±0.40
RH morning** (%)	52.52±3.33	87.34±0.47
RH evening** (%)	62.34±2.74	86.16±0.88
Mean RH** (%)	57.43±2.79	86.75±0.55
THI morning**	46.10±0.35	59.20±0.30
THI evening**	48.69±0.44	63.81±0.46
Mean THI**	47.40±0.37	61.51±0.35

** p<0.01, means differed significantly. RH=Relative humidity, THI=Temperature and humidity index

The HR during the morning were 67.21±0.28, 64.44±1.06 and 67.17±0.15 per min, respectively, for the cows in the T_1_, T_2_, and T_3_; the difference between the treatments were statistically (p>0.05) comparable for overall mean. Similarly, the overall mean HR values at the afternoon were 61.58±1.34, 60.67±1.06 and 59.40±0.98 per min for the cows in the T_1_, T_2_, and T_3_, respectively, and the difference between treatments overall mean of afternoon were also statistically comparable (Table-2).

**Table-2:** Effect of drinking water temperature on physiological variables such as HR, RR, RT and RM at forenoon and afternoon.

Attributes	T_1_	T_2_	T_3_
HR (beats/min) at forenoon^NS^	67.21±0.28	64.44±1.06	67.17±0.15
HR (beats/min) afternoon^NS^	61.58±1.34	60.67±1.06	59.40±0.98
RR (beats/min) forenoon^NS^	20.44±0.71	22.40±0.88	20.48±0.59
RR (breaths/min) at afternoon^NS^	23.88±1.03	26.13±1.02	24.75±0.76
RT (°F) at forenoon*	100.17±0.11^b^	100.46±0.12^ab^	100.65±0.10^a^
RT (°F) at afternoon**	100.16±0.12^b^	100.81±0.08^a^	100.68±0.08^a^
RM (motility/3 min) at forenoon^NS^	4.54±0.18	4.48±0.19	4.10±0.16
RM (motility/3 min) at afternoon**	4.46±0.16^a^	4.56±0.19^a^	3.56±0.15^b^

** p<0.01= means among the treatments are statistically different and significant at 1% level,

* p<0.05 = means among the treatments are statistically different and significant at 5% level, means having different superscription in row differ significantly.

NS=Statistically nonsignificant, HR=Heart rate, RR=Respiration rate, RT=Rectal temperature, RM=Ruminal motility, NS=means among the treatments are statistically comparable at 5% level

The result indicated that at low ambient temperature (mean daily temperature of 11.64±0.40°C and range of 0-14°C adult crossbred cow maintained normal physiological HR and providing warm drinking water did not influence the HR. The observation is in agreement with that of McDonald and Bell [14] who concluded that low temperature *per se* do not affect HR changes in lactating, mature cows that have been acclimatized to low temperatures and high humidity.

The RR during the morning were 20.44±0.71, 22.40±0.88 and 20.48±0.59 per min, respectively, for the cows in the T_1_, T_2_, and T_3_; the difference between the treatments were statistically (p>0.05) comparable for overall mean (Table-2). Similarly, the overall mean RR values at the afternoon were 23.88±1.03, 26.13±1.02 and 24.75±0.76 per min for the cows in the T_1_, T_2_, and T_3_, respectively, and the difference between treatments overall mean of afternoon were also statistically comparable (Table-2). The result indicated that at low ambient temperature (mean daily temperature of 11.64±0.40°C and range of 0-14°C) adult crossbred cow maintained normal physiological RR and providing warm drinking water did not influence the HR. The observation is also in agreement with that of McDonald and Bell [14] who concluded that low temperature *per se* does not affect RR changes in lactating, mature cows that have been acclimatized to low temperatures and high humidity.

RT at morning was significantly (p<0.05) higher in 35-40°C warm drinking water groups T_3_ than cows in T_2_ and T_1_ groups while the difference in RT among T_2_ and T_1_ was statistically comparable (Table-2). The overall mean RT of cows at morning in T_1_, T_2_, and T_3_ were 100.17±0.11°F, 100.46±0.12°F, and 100.65±0.10°F, respectively. Similarly, RT at evening was significantly higher for warm drinking water group’s cow (T_2_ and T_3_) than cow provided with ambient cool drinking water (T_1_). However, overall mean of RT at evening was significantly (p<0.01) higher for cows consuming warm drinking water in T_2_ (15-20°C) and in T_3_ (35-40°C) than cows consumed ambient cool drinking water T_1_. The overall mean RT at evening in T_1_, T_2_, and T_3_ were 100.16±0.12°F, 100.81±0.08°F, and 100.68±0.08°F, respectively. The observation indicated that variation in RT between across the experiment did not show a definite trend with ambient temperature and providing warm drinking water did influence the body temperature.

According to Degen and Young [15] there was a greater decrease in rumen temperature, and it took longer to recover with animal consuming the snow (−10°C and ice 0°C) treatments than with cold water (0°C) and warm water (30°C) treatments. Changes in RT were delayed relative to the changes in rumen temperature in all treatments; maximum decrease in RT were 1.4°C for the ice, 1.1°C for the snow, 0.5°C for the cold water and 0.4°C for the warm water. By increasing the temperature of deep tissue, the animal is able to maintain homeothermy in vital organs which would otherwise be upset by the return of chilled blood from periphery. Body heat losses, particularly by conduction and convection, are also reduced to minimum. Lefcourt and Adams [16] found that ambient temperature affected body temperature when a certain threshold was attained. These effects are attributed to the water’s refreshing capacity which facilitates heat dissipation and helps to decrease metabolic load [17]. These findings are exactly contrast with Savage *et al*. [18] who reported that increase in the average RT of the sheep when they received water at 0°C and 10°C compared with water at 30°C.

The RM at morning and evening is presented in (Table-2). The overall mean of the RM during morning were 4.54±0.18, 4.48±0.19, and 4.10±0.16 per 3 min, respectively, for the cows in the T_1_, T_2_, and T_3_, the differences among the treatments were non-significant. The corresponding values at the afternoon were 4.46±0.16, 4.56±0.19 and 3.56±0.15 per 3 min. The rumen motility at evening was significantly higher for cows consumed ambient cool drinking water (T_1_) and cows consuming 15-20°C water (T_2_) than for cows consuming warm drinking water in T_3_ (35-40°C). The result indicated that low ambient temperature and providing cool drinking water increased the rumen motility, and it was corroborated with findings of Dikmen *et al*. [19], Graunke *et al*. [20].

According to Atterbery and Jhonson [21] a significant (p<0.01) decrease was noted in the amplitude of the rumen contractions when animals were exposed to 38°C. Feedlot cattle exposed to cold temperature showed increased rumination activity [22]. When cattle were exposed to cold stress, gastrointestinal tract motility increased due to an elevated metabolic rate, resulting from an increase in concentration of thyroid hormone and its activity for to meet high energy demands to maintain core body temperature [23].

## Conclusion

Provision of warm water 35-40°C to dairy crossbred cows not only helped to conserve the energy but also maintained the normal RM during winter season at high altitude temperate region. Therefore, it can be concluded that offering warm drinking water at 35-40°C to crossbred lactating dairy cow is beneficial during winter at the high altitude temperate region.

## Authors’ Contributions

DMG did the physical experiment. PT designed the work, and BHMP guided the experiment. VKU along with SS did the statistical analysis. GKG and SHB revised the manuscript. All authors read and approved the final manuscript.
